# Preparation and characterization of multiphase ceramic designer waste forms

**DOI:** 10.1038/s41598-021-84014-1

**Published:** 2021-02-25

**Authors:** Braeden M. Clark, Priyatham Tumurugoti, Shanmugavelayutham K. Sundaram, Jake W. Amoroso, James C. Marra

**Affiliations:** 1 Multifunctional Materials Laboratory (M&M) Lab, Inamori School of Engineering, The New York State College of Ceramics, Alfred University, Alfred, NY 14802 USA; 2 Savannah River National Laboratory, Aiken, SC 29808 USA

**Keywords:** Ceramics, Nuclear waste

## Abstract

The long-term performance, or resistance to elemental release, is the defining characteristic of a nuclear waste form. In the case of multiphase ceramic waste forms, correlating the long-term performance of multiphase ceramic waste forms in the environment to accelerated chemical durability testing in the laboratory is non-trivial owing to their complex microstructures. The fabrication method, which in turn affects the microstructure, is further compounding when comparing multiphase ceramic waste forms. In this work, we propose a “designer waste form” prepared via spark plasma sintering to limit interaction between phases and grain growth during consolidation, leading to monolithic high-density waste forms, which can be used as reference materials for comparing the chemical durability of multiphase waste forms. Designer waste forms containing varying amounts of hollandite in the presence of zirconolite and pyrochlore in a fixed ratio were synthesized. The product consistency test (PCT) and vapor hydration test (VHT) were used to assess the leaching behavior. Samples were unaffected by the VHT after 1500 h. As measured by the PCT, the fractional Cs release decreased as the amount of hollandite increased. Elemental release from the zirconolite and pyrochlore phases did not appear to significantly contribute to the elemental release from the hollandite phase in the designer waste forms.

## Introduction

The most widely studied ceramic waste forms are derived from SYNROC (i.e., synthetic rock) materials developed in Australia in the late 70’s^1^. SYNROC-type phases are based on titanate minerals and are attractive for high-level waste (HLW) immobilization due to their incorporation for nearly all elements present in HLW into a crystalline lattice. More generally, crystalline waste forms are of interest because they provide the possibility of higher waste loading and superior chemical durability compared to glass waste forms^2,3^. Characteristic titanate minerals comprising SYNROC include hollandite, zirconolite, pyrochlore, and perovskite phases. Elements with a 3+ or 2+ valance states form perovskite-((A^+2^)TiO_3_) and pyrochlore ((A^+3^)_2_Ti_2_O_7_)-type phases while zirconium (4+ valence) partitions to a zirconolite (CaZrTi_2_O_7_) phase. Zirconolite and pyrochlore are the major immobilization hosts for actinides and lanthanides. Cs and other alkali metals partition to a hollandite structure based on the general formula Ba_x_Cs_y_M_z_Ti^+4^_8−z_O_16_, where z = 2x + y for trivalent M cations^4–9^.

The chemical durability of nuclear waste form determines elemental release and defines the long-term performance of the waste form. However, durability is not an intrinsic material property but, is influenced by controlled parameters during testing. Tests can be conducted with varying surface area to volume ratios (SA/V) to accentuate solution chemistry or material chemistry. The most widely used test methods for studying aqueous leach behavior include the product consistency test (PCT) (ASTM C1285)^5,10,11^, the Materials Characterization Center (MCC)-1 and MCC-3 standards^12–14^, the vapor hydration test (VHT) (ASTM C1663)^15–17^, and the single-pass flow-through (SPFT)^18^. The MCC-1 and VHT tests use a monolith sample, whereas the MCC-3 and PCT tests use a crushed sample powder with a standard surface area on the order of 2000 m^−1^. These tests are static tests and are intended for homogenous glass waste forms or systems in which a homogeneous glassy phase controls the leach behavior. The SPFT is a dynamic test used to measure the dissolution rate and was also developed for homogeneous glass waste forms. Standard tests have not been developed exclusively for determining the durability of materials with more complex microstructures like multiphase crystalline ceramics.

A well-controlled chemical durability measurement for multiphase ceramic waste forms that enables correlation of the long-term performance is challenging due to the complexities inherent in the microstructures of crystalline materials. Moreover, the variations in chemistry and processing methods can contribute to differences in the chemical durability. One method to overcome many of the inherent testing challenges is to process and characterize the phases individually as single phase ceramics. However, this is time consuming, likely prohibitively expensive, and does not account for mixed phase interactions that would be present in monolithic multiphase ceramic waste forms.

We report use of spark plasma sintering (SPS) as a method to produce ceramics with uniform and reproducible microstructures. SPS can produce near-net shape samples that can be tailored with varying volumes of constituent phases. The volume ratios can be readily changed to elucidate the effects of the different phases on the chemical durability of the waste form. The short sintering times that can be achieved during SPS were intended to limit reaction between the component phases. The SPS method as described in this work, has been used to achieve samples with higher theoretical density in a shorter processing time and minimal grain growth compared to conventional sintering methods. Recently, SPS has been heavily investigated as a process for fabricating waste forms^19–24^, including the phases of interest to the work presented here: hollandite^25^, zirconolite^26,27^, and pyrochlore^28^.

The multiphase ceramics produced have been designed with specific volume fractions of the constituent phases and exhibit consistent, uniform microstructure. Our work presented demonstrates the usefulness of the methodology to develop a set of protocols to develop a multiphase ceramic with tailored microstructure that can be used for systematic comparisons among different research groups. It should also be noted that processing will affect the radiation durability as seen previously^29^, however the focus of this paper is on chemical durability.

## Experimental materials and methods

### Methodology

**Figure 1:**
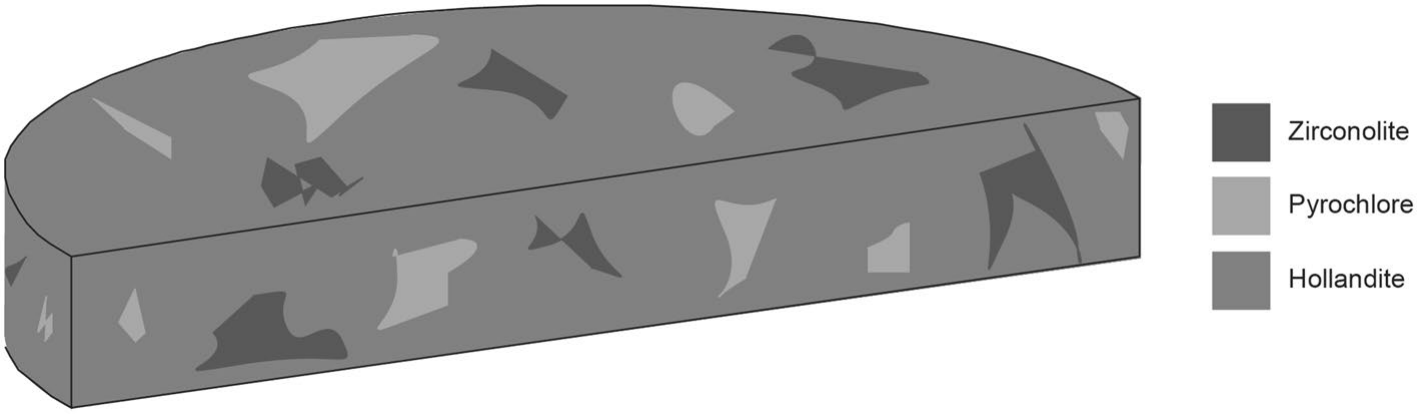
Schematic of a designer waste form.

An idealized multiphase ceramic waste form targeted in this work is schematically shown in Fig. 1. The majority phase (> 50% typically) in the systems presented is hollandite. Hollandite, which is the host for cesium, is historically the most troublesome phase to engineer. As such, multiphase ceramic compositions were developed based upon single phase hollandite composition development. Simulated HLW compositions were developed by the Savannah River National Laboratory (SRNL) based on waste stream projections for reprocessing used nuclear fuel (UNF) from commercial reactors under the auspices of the US Department of Energy’s (DOE) Fuel Cycle Research & Development program. The compositions, listed in Table 1 were based on previous work and contain Cr as an additive to promote stable hollandite formation, as detailed elsewhere^30^. Single phase hollandites designated with the predecessors Cr–Al–Fe and Cr have compositions of BaCs_0.3_CrAl_0.3_FeTi_5.7_O_16_ and BaCs_0.3_Cr_2.3_Ti_5.7_O_16,_ respectively and are incorporated into their corresponding multiphase compositions.

**Table 1:** Single-phase and multiphase simulated waste form compositions (wt %).

Sample ID/oxide	Cr–Al–Fe hollandite	Cr hollandite	Multiphase Cr–Al–Fe hollandite	Multiphase Cr hollandite
Al_2_O_3_	1.86	–	1.27	–
BaO	18.65	18.57	12.76	12.72
CaO	–	–	1.39	1.38
Cr_2_O_3_	9.25	21.17	6.33	14.5
CdO	–	–	0.11	0.11
Ce_2_O_3_	–	–	3.10	3.09
Cs_2_O	5.14	5.12	2.88	2.87
Eu_2_O_3_	–	–	0.17	0.17
Fe_2_O_3_	9.71	–	6.65	–
Gd_2_O_3_	––	–	0.16	0.16
La_2_O_3_	–	–	1.58	1.58
MoO_3_	–	–	0.85	0.84
Nd_2_O_3_	–	–	5.23	5.22
Pr_2_O_3_	–	–	1.45	1.44
Rb_2_O	–	–	0.42	0.42
SeO_2_	–	–	0.08	0.08
Sm_2_O_3_	–	–	1.08	1.07
SnO_2_	–	–	0.07	0.07
SrO	–	–	0.98	0.98
TeO_2_	–	–	0.66	0.65
TiO_2_	55.38	55.14	49.16	49.01
Y_2_O_3_	–	–	0.63	0.63
ZrO_2_	–	–	2.99	2.98

Both single phase hollandite and multiphase (hollandite, zirconolite, and perovskite) samples were prepared. Single phase hollandite and their corresponding multiphase compositions (i.e. compositions incorporating respective hollandite stoichiometry) were prepared via sintering and SPS in order to compare processing technologies. The designer waste forms were prepared by SPS of volumetric mixtures of pre-reacted single phase constituents (i.e. zirconolite, pyrochlore, and hollandite).

#### Single phase hollandite and multiphase Cr–Al–Fe and Cr–Hollandite

Oxide and carbonate precursors were mixed together in stoichiometric amounts to target the composition listed in Table 1. The powders were ball milled with deionized water using zirconia media in a polyethylene jar for 2 h. The slurry was dried overnight and then separated from the media. The blended powders were then subjected to solid state reaction or SPS processes. Solid state reaction of single phase and multiphase ceramic waste forms was performed in a tube furnace by placing loosely packed batch powders into an alumina crucible and heating the furnace to 1500 °C at a rate of 5 K/min, holding for 0.5 h and allowing to furnace cool to room temperature. These parameters have been shown to obtain the phases desired, while limiting the hold time to prevent elemental volatilization^25^. Densification via SPS was carried out using a FCT HP D 25 (FCT Systeme GmbH, Rauenstein, Germany) furnace with graphite dies and punches. The unreacted powder was placed inside of the graphite die with a thin layer of graphite paper between the die and sample. The reaction schedule was as follows: a heating rate of 100 °C/min to a maximum temperature of 1000 °C for Cr–Al–Fe hollandite or 1125 °C for Cr hollandite, a hold time for 3 min, and a cooling rate of 100 °C/min. A uniaxial pressure of 54 MPa was applied throughout the densification process.

#### Designer waste forms

Designer waste forms were prepared from volumetric mixtures of pre-reacted single phase hollandite, zirconolite, and pyrochlore. Single phase hollandite (Ba_1.1_Cs_0.1_Cr_2.3_Ti_5.7_O_16_ or Ba_0.9_Cs_0.5_Cr_2.3_Ti_5.7_O_16_), zirconolite (CaZrTi_2_O_7_), and pyrochlore (Nd_2_Ti_2_O_7_) were synthesized via solid state reaction. Hollandite precursor powders (BaCO_3_, Cr_2_O_3_, and TiO_2_) were milled together in a high-density polyethylene (HDPE) bottle with zirconia media and an ethanol/water mixture for 1 h. The media was separated and the slurry was dried on a hot plate at 100 °C. Zirconolite precursor powders (CaO, ZrO_2_, and TiO_2_) were milled using a ZrO_2_ jar with 2 mm yittria-stabilized zirconia (YSZ) media at 1,200 rpm using a mixture of ethanol/water in a VQ-N High Speed Ball Mill (Across International) for 0.25 h. Pyrochlore precursors of (Nd_2_O_3_ and TiO_2_) were milled in a HDPE jar with ¼ inch spherical alumina media in an ethanol/water mixture for one h. The resulting powders were used to form 20 mm diameter pellets using a steel die set and hydraulic press. The pellets for hollandite were reacted in a furnace at 1500 °C for 0.5 h on Pt foil. The pellets for zirconolite were reacted in a furnace at 1350 °C for 48 h on a Pt foil. The pellets for pyrochlore were reacted in a furnace at 1400 °C for 24 h, crushed in an alumina mortar and pestle, re-pelletized, and reacted a second time at 1400 °C for 24 h on a Pt foil.

The single-phase powders described above were mixed together in various amounts after the resulting pellets were crushed and ground to target the designer waste form mixtures listed in Table 2 by milling in a HDPE jar with zirconia media and ethanol/water for 1 h. The multiphase powder mixtures were subsequently dried and sintered using SPS. The mixed powders were loaded into a graphite die (inner diameter of 18.75 mm) lined with graphite foil to prevent interaction between the powders and the die. The samples were heated at 100 °C/min to a maximum temperature of 1100–1133 °C and held at temperature for 30 s. The samples were cooled to room temperature at 100 °C/min. A pressure of 54 MPa was applied at room temperature and held during heating and holding and slowly released upon cooling. The designations ‘hollandite high cesium’ and ‘hollandite low cesium’ correspond to high or low cesium content in the hollandite phase. The zirconolite to pyrochlore ratio is similar to that found in SYNROC compositions.

**Table 2:** Phase composition (vol. %) of the designer waste form target compositions.

Sample ID	Phase (Vol. %)		
	Hollandite Ba_1.1_Cs_0.1_Cr_2.3_Ti_5.7_O_16_	Zirconolite CaZrTi_2_O_7_	Pyrochlore Nd_2_Ti_2_O_7_
80%-hollandite low cesium	80	11.75	8.25
60%-hollandite low cesium	60	23.5	16.5
40%-hollandite low cesium	40	35	25
20%-hollandite low cesium	20	47	33

Sample ID	Hollandite Ba_0.9_Cs_0.5_Cr_2.3_Ti_5.7_O_16_	Zirconolite CaZrTi_2_O_7_	Pyrochlore Nd_2_Ti_2_O_7_
80%-hollandite high cesium	80	11.75	8.25
60%-hollandite high cesium	60	23.5	16.5
40%-hollandite high cesium	40	35	25
20%-hollandite high cesium	20	47	33

### *Leach* Testing

The PCT Method-A^10^ was performed on each composition to assess chemical durability. As-synthesized samples were ground to 100–200 mesh particle size, washed and prepared according to the standard procedure (ASTM C1285). The chemical composition of the as-synthesized samples was measured using inductively coupled plasma (ICP)—atomic emission spectroscopy (AES) and—mass spectrometry (MS) for Cs. Fifteen milliliters of water was added for 1.5 g of sample in stainless steel vessels. Samples were measured in triplicate except for 20%-hollandite high cesium, which was measured in duplicate. The vessels were sealed and placed in an oven at 90 ± 2 °C for 7 days. Once cooled, the resulting solutions were acidified and analyzed for cation concentrations using ICP-AES and MS. The elemental release is reported as the fractional release rate of element i in each specimen (*FR*_*i*_) and was determined according to the following equation:

1 $$ FR_{i} = \frac{{C_{i} V_{s} }}{{f_{i} m_{s} }} $$

where *C*_*i*_ is the concentration of element i in the leachate (g/L), *V*_*s*_ is the leachate volume (L), *f*_*i*_ is the fraction of element i in the as-processed, unleached specimen (unitless), and *m*_*s*_ is the sample mass (g). Uncertainty in the reported values were calculated based on + /− 20% (2σ) error in the ICP measurements.

**Figure 2:**
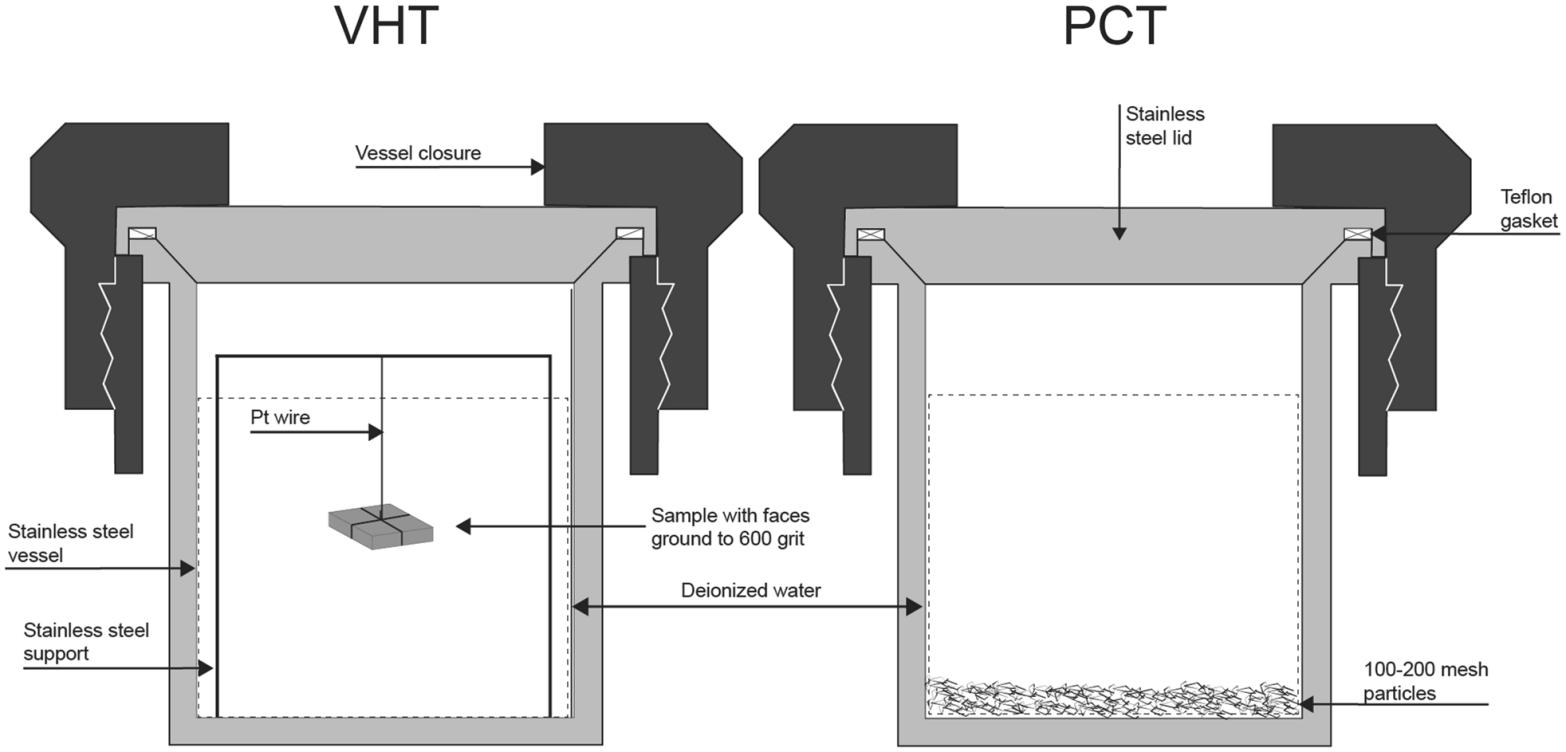
Schematic of the VHT and PCT sample set up.

Monolithic samples of the four low cesium hollandite compositions were subjected to vapor hydration testing (VHT)^15^. The approximate sample dimensions were 10 × 10 × 2 mm. Each sample face was ground to 600 grit with SiC paper and suspended from a stainless steel support with Pt wire. Figure 2 shows a schematic of the sample set up. Suspended samples were placed and sealed in stainless steel vessels with 0.25 mL of water. The vessels were placed in an oven at 200 °C for 1500 h. Vessel weights were periodically checked for water loss and replenished if > 0.05 ml was lost. At no time during the testing were the vessels dry. After testing, the samples were analyzed with XRD, then sectioned and polished, and examined with SEM–EDS.

## Results and discussion

### Single phase hollandite and multiphase Cr–Al–Fe and Cr–Hollandite

**Figure 3:**
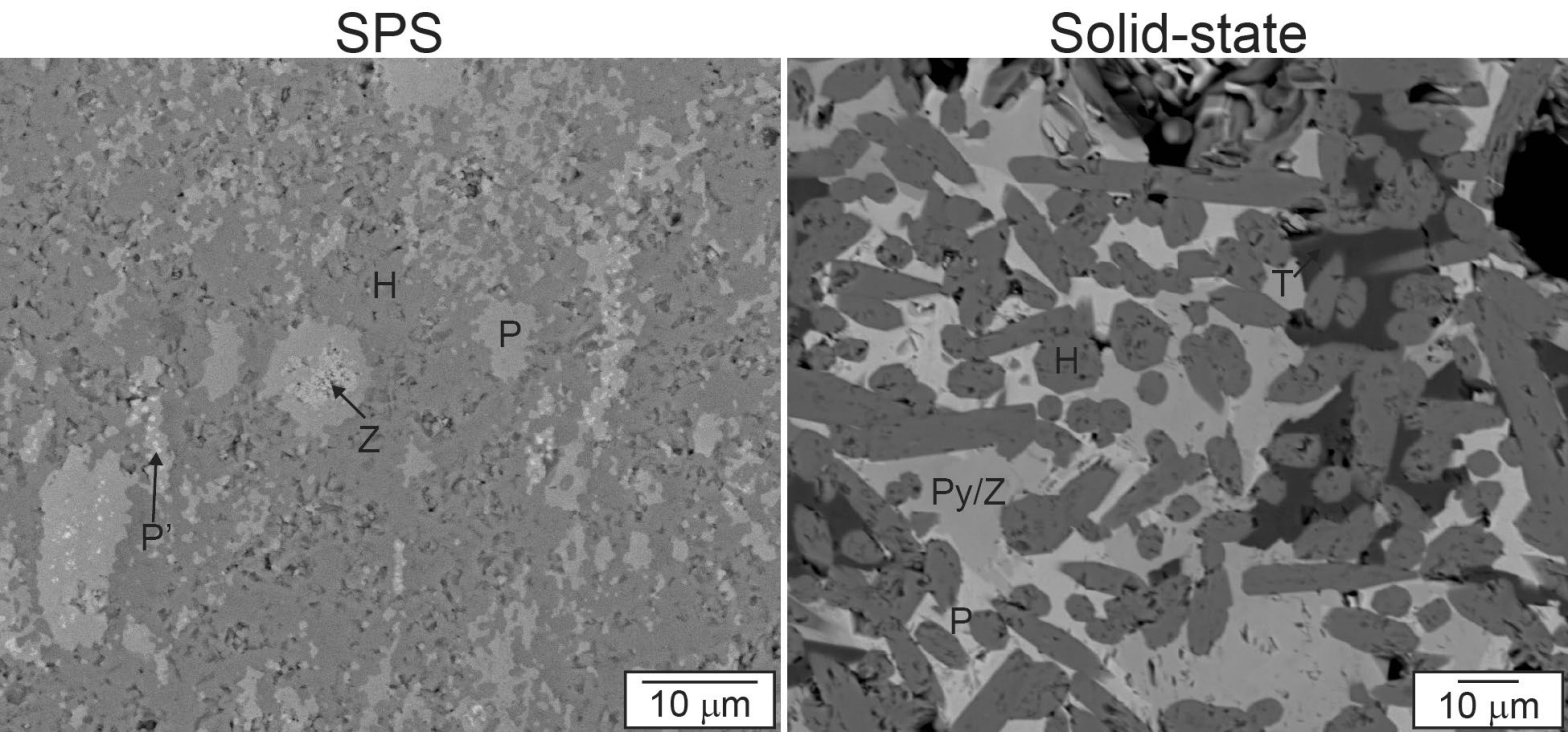
Microstructures of SPS and solid-state sintered surrogate ceramic waste forms, H—hollandite, P and P’—perovskite, zirconium-rich—Z, TiO_2_—T, pyrochlore/zirconolite—Py/Z.

The microstructure and phase assemblages of single phase and multiphase sample have been described previously^31^. In general, the SPS method produces a fine-grained microstructure, whereas the microstructure of solid-state produced samples exhibit larger grains. Examples of the microstructure of the multiphase Cr–hollandite samples processed by solid-state sintering and SPS are shown in Fig. 3 for comparison. In both processes, the target phases, hollandite, zirconolite, pyrochlore, and perovskite were observed by XRD and are identified in the phase contrast images shown in Fig. 3. However, the microstructures produced are complex as the phases can adopt a wide range of chemical substitution and multiple chemistries (with some being more favorable than others) of the same structure are known to exist in a single sample. As can be seen in Fig. 3, in the SPS sample, hollandite formed the matrix, while two perovskite phases and a zirconium-rich phase (zirconolite/pyrochlore) were dispersed throughout. In contrast, sintered samples exhibited elongated hollandite grains dispersed with TiO_2_, pyrochlore/zirconolite, and perovskite phases.

**Figure 4:**
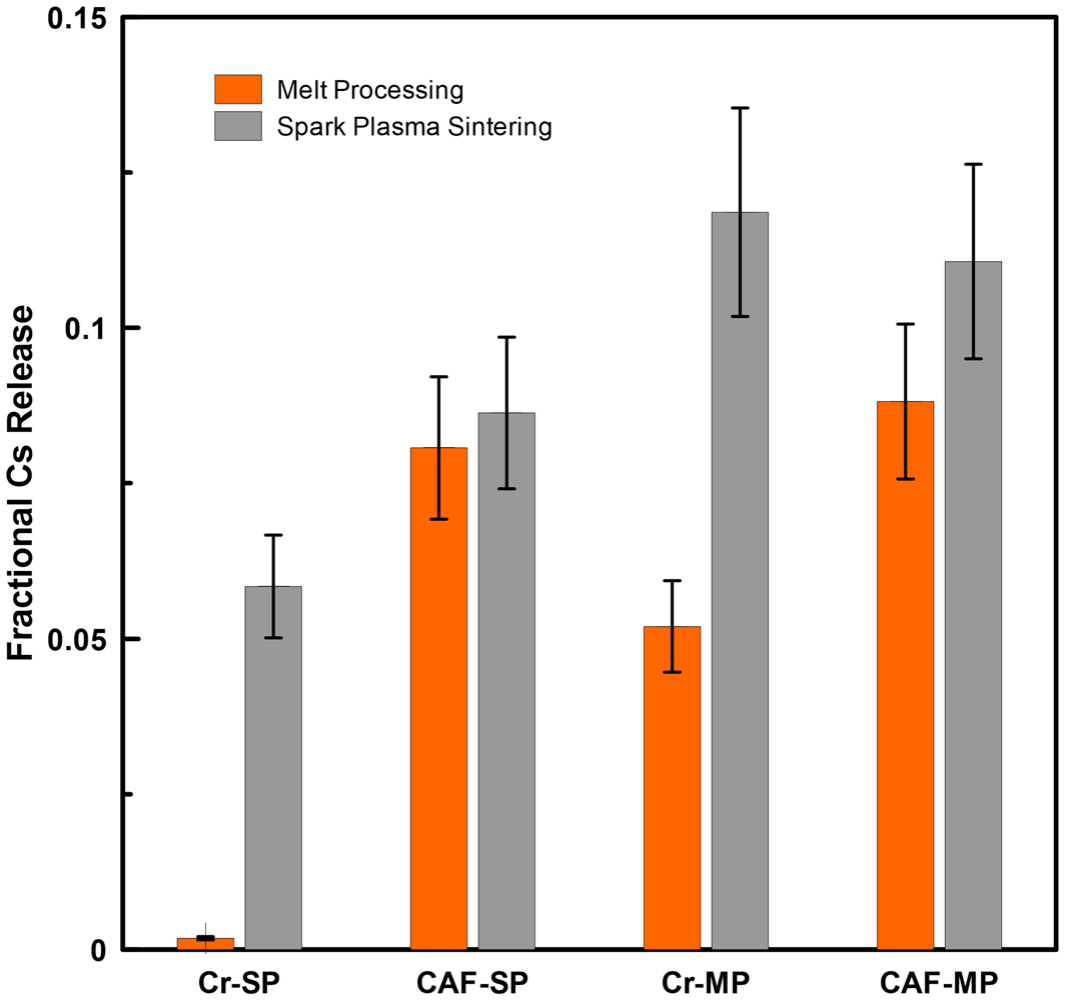
Comparison of fractional Cs release across single phase (SP) and multiphase (MP) ceramics targeting Cr–Al–Fe (CAF) and Cr hollandite compositions prepared via melt-processing and SPS.

The fractional release of Cs from the single-phase Cr–Al–Fe and Cr hollandites and the corresponding multiphase reference materials is presented in Fig. 4. SPS samples exhibited a greater fractional Cs release compared to the melt processed samples. It should be re-iterated that the Cs release was normalized to the measured composition, so volatilization of species would not affect the results. In Fig. 4, a significant difference in Cs elemental release was observed between SPS and solid state sintering, indicating that processing can affect the leach behavior in multiphase ceramic systems. This result is not unexpected considering that surface area and microstructural features significantly affect elemental release in the PCT and other accelerated chemical durability tests. The rapid release of Cs has been noted previously and has been attributed to soluble Cs phases generated during processing^32^ and in batch materials^33^. The Cr and Cr–Al–Fe based hollandites chosen for these experiments have been shown to prevent these Cs soluble phases to develop during melt processing^30^. It was shown in previous results^34^ that a Cs rich phase is formed during the SPS process, which would account for the higher release values of the SPS samples during the relatively short PCT experiment. Alternative processing SPS conditions/parameters need to be explored in order to eliminate these soluble Cs phases. Additionally, although the material in this work was sieved and size fractioned to obtain a reproducible and consistent surface area, the geometric surface area estimated in that manner does not explicitly take into account microstructural features such as voids (smaller than the sieve fraction size) particle anisotropy, or irregular (non-spherical) surface areas, which could affect the leaching behavior. To the best of our knowledge, no systematic quantitative method has been reported in the literature on evaluating waste forms with identical stoichiometry and phase assemblage but distinctively different microstructure, similar to a multiphase ceramic material. Therefore, the designer waste form was explored as a method to systematically study chemical leaching from ceramic waste forms. Specifically, the intent was to fabricate highly dense multiphase ceramics with comparable microstructure and phase assemblages (i.e., known ratios of phases).

### Designer waste forms

**Figure 5:**
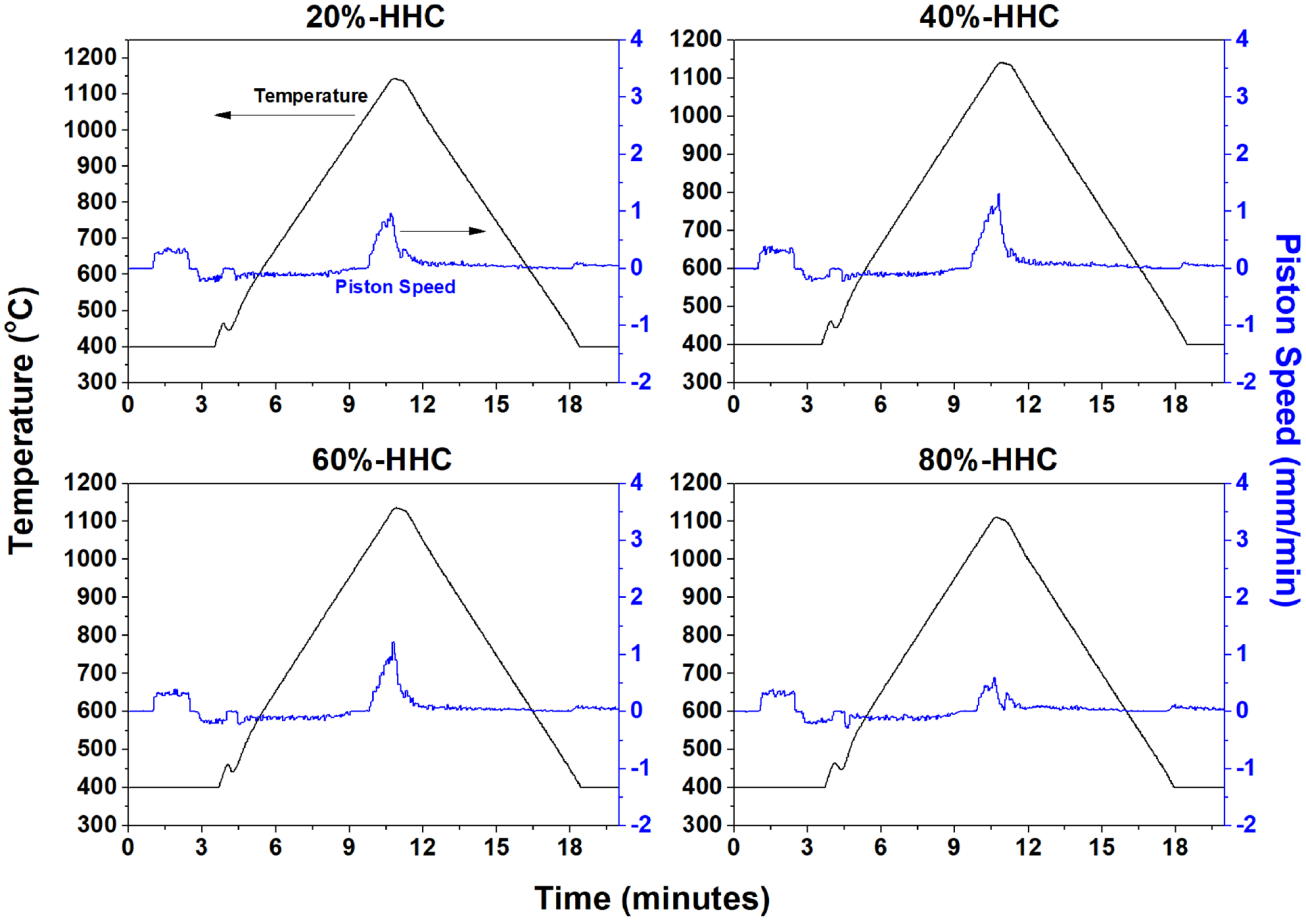
SPS densification curves of high Cs content hollandite composition (HHC) designer waste form compositions.

SPS was selected as a viable method to produce the consistent and comparable microstructures needed in the designer waste forms. The sintering curves displaying the temperature and piston speed as a function of time of the high Cs content hollandite compositions are shown in Fig. 5. The low Cs content hollandite composition curves are similar and therefore are not shown. Consolidation of the materials occurs immediately prior reaching maximum temperature. The maximum temperature was slightly reduced with increasing hollandite content due to material ejecting from the die at higher hollandite content.

**Figure 6:**
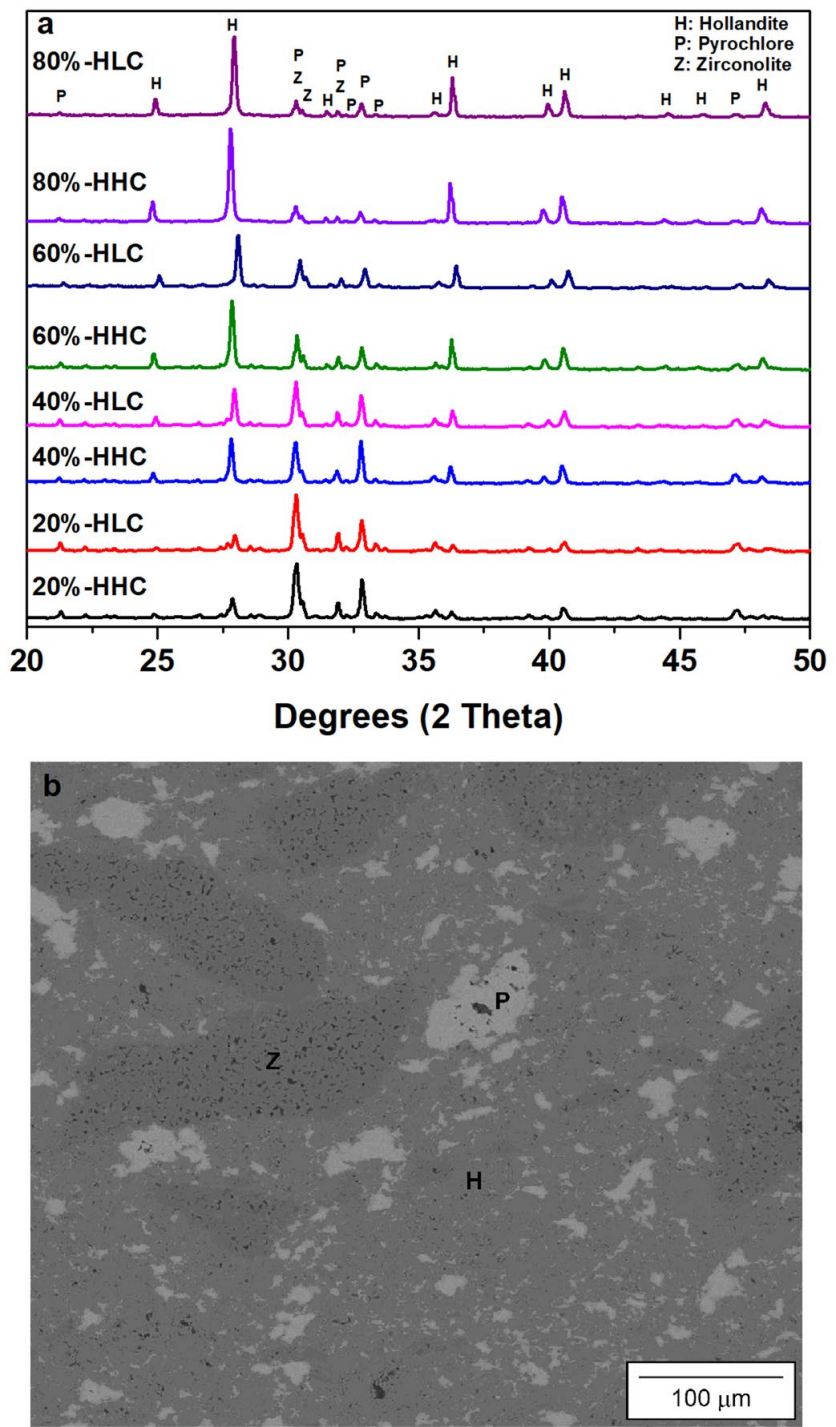
XRD patterns of designer waste forms fabricated using both high (HHC) and low (HLC) Cs content hollandite compositions (**a**) and the microstructure of 60%-HLC (**b**).

X-ray diffraction (XRD) was performed on powdered samples to determine if any intermediate phases formed during SPS. The patterns of all compositions are displayed in Fig. 6. A slight shift towards lower 2 theta is seen in the hollandite peaks in the samples with higher Cs content as would be expected when substituting Cs for Ba^35^. All the diffraction peaks could be identified as belonging to hollandite, zirconolite, or pyrochlore phases. A representative microstructure from the 60%-hollandite low Cs sample after processing is displayed in Fig. 6. The matrix is hollandite phase with distinct regions of varying zirconolite and pyrochlore as described previously. The microstructures of the other designer waste form compositions (80, 40 and 20%-hollandite) look similar to the displayed image but with volumetric amounts of the phases changing according to the batched composition.

**Figure 7:**
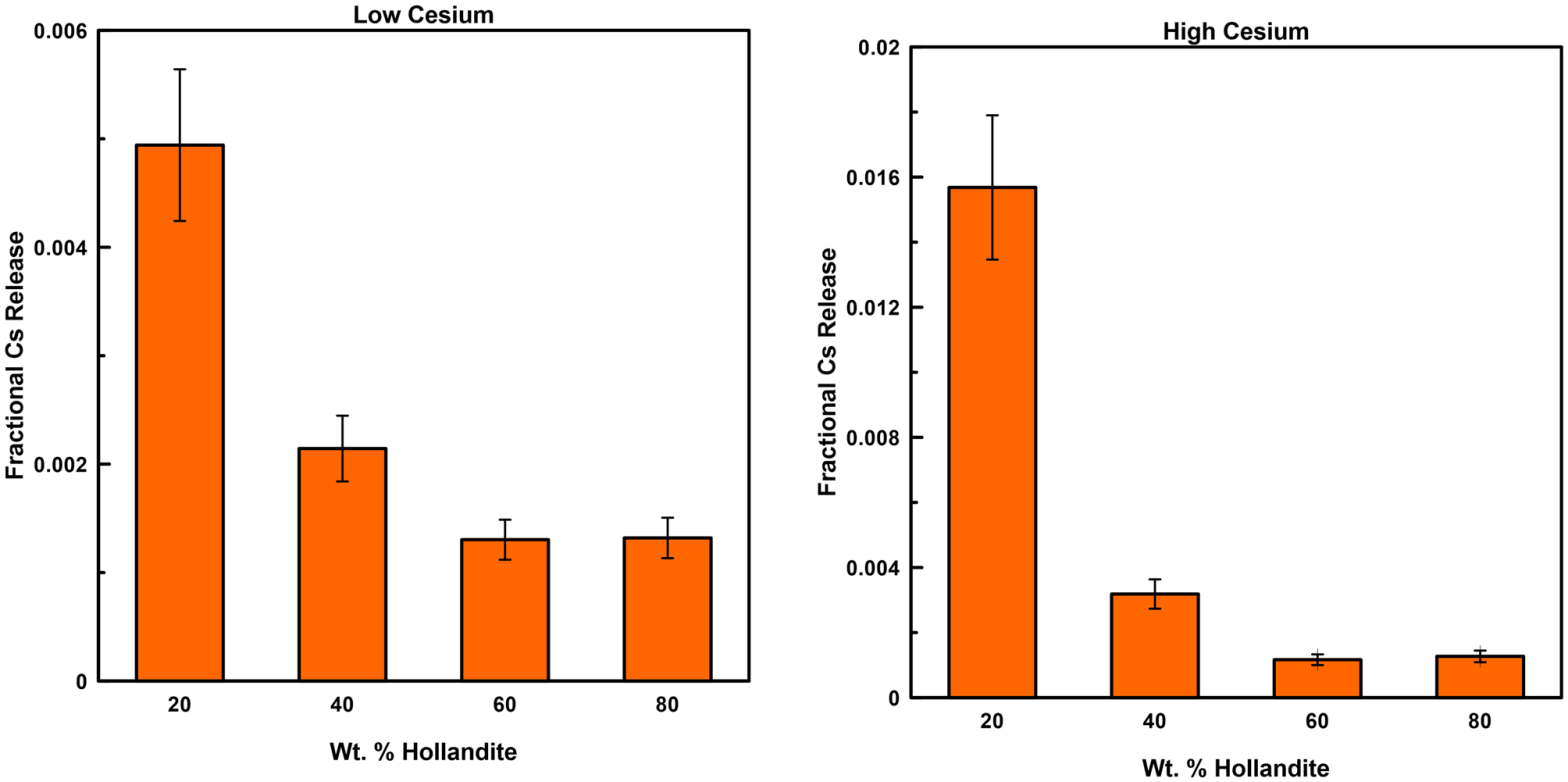
Normalized Cs release from both low and high Cs containing hollandite designer waste form compositions.

The PCT was performed on designer waste form samples and fractional Cs release from the samples as a function of hollandite content is presented in Fig. 7. For all hollandite concentrations, the higher Cs content samples released a greater percentage of the total Cs than their lower Cs content counterparts. This result could be explained by relative concentrations of Cs available for leaching, but the fractional Cs release was measured to decrease as the amount of hollandite increased in both series of samples and recent studies indicate that increasing Cs concentration in the hollandite phase can decrease the fractional Cs release^36^. In this work, the increasing hollandite content, relative to the secondary phase, appeared to impart the same effect of decreasing Cs release. It is possible that secondary phases competing for the Cs could result in hollandite forming with a Cs deficiency, a scenario that would be amplified in samples with lower total hollandite percentage. However, while parasitic Cs-rich phases with lower durability were not observed using the SEM or XRD, based on measured composition data, the low Cs content hollandite samples contained 31.2 wt% +/− 1.9 relative standard error (%) of the batched Cs whereas the high Cs content hollandite samples contained 57.2 wt% +/− 1.7 relative standard error (%). This apparent inverse relationship between Cs loss and Cs content in the hollandite is not uncommon and further suggests thermodynamic factors linked to the Cs content. Because the fraction Cs release was normalized to the measured compositions after grinding and sieving to represent the true leach sample, it is conceivable that the low Cs content hollandite samples may have formed Cs-rich phases during synthesis that were subsequently washed/leached during preparation of the PCT samples. If indeed the durability (as measured by Cs release) of hollandite is correlated to the Cs content, then by extension the stability of the hollandite would be affected similarly. It follows that the low Cs content hollandite samples, if prepared with a sub-optimal Cs content and being thermodynamically less stable than the high Cs content hollandite samples, could result in both parasitic Cs-rich phases (i.e. at grain boundaries) as well as a significant fraction of the hollandite phase being poorly formed. As a result, the majority of the Cs remaining after synthesis was leached during the preparation steps and does not represent a comparable sample. No matter the cause, the leach results confirm the complexity and challenges associated with comparing chemical leach results across multiphase waste forms and re-enforce the need for reference materials like designer waste forms.

**Figure 8:**
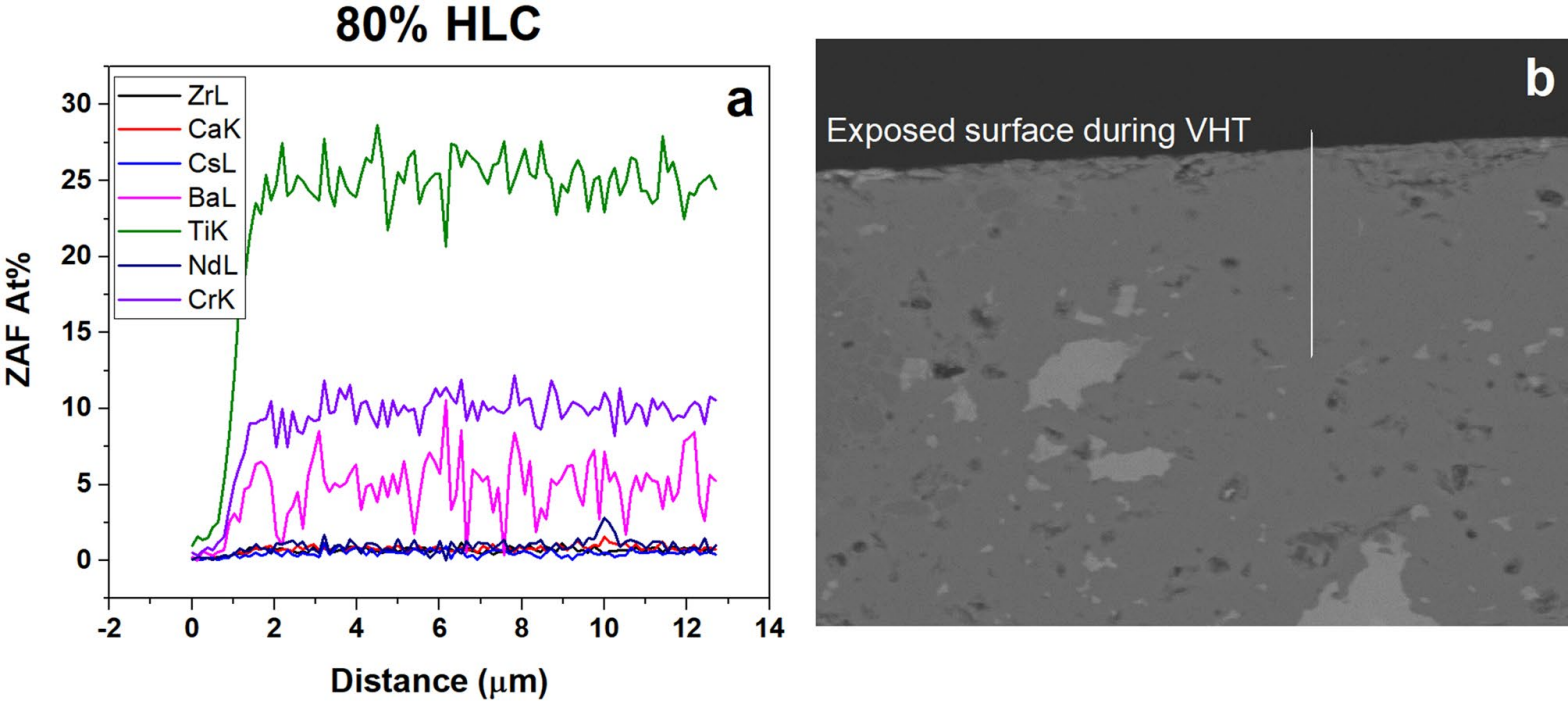
(**a**) SEM–EDS line scan results across the hollandite phase of the 80%-hollandite low Cs (HLC) designer waste form sample and (**b**) corresponding SEM image.

VHT was conducted on low Cs content hollandite compositions to provide complementary information about behavior of these materials at elevated temperature in a saturated (wet) atmosphere. After 1500 h at 200 °C, the sample surfaces appeared to be unaffected. By XRD, only a diffraction peak belonging to Pt developed during testing. No discernable surface degradation or chemical changes was observed with SEM or EDS in any of the samples subjected to VHT. SEM–EDS line scans across the hollandite phase at the surface of the 80%-hollandite low Cs content sample are shown in Fig. 8. Line scans were performed ~ 12–15 μm from the surface into the sample. No significant chemical gradient could be discerned, indicating that elemental diffusion was not significant, under these accelerated degradation conditions. Both C and O are removed from the EDS results.

## Conclusions

SPS was used to fabricate designer waste forms with controlled microstructure and phase composition. Microstructural characterization and phase analysis show that SPS can be used to consolidate multiphase materials into monolithic, dense designer waste forms. Multiphase ceramic designer waste forms consisting of the typical SYNROC phases were prepared and evaluated for their chemical durability using the VHT and PCT procedures. Analysis of samples after the VHT indicated excellent chemical stability of the designer waste forms under saturated atmospheres at elevated temperatures as evidenced by microstructural and chemical analysis. The fractional Cs release, as measured by the PCT, decreased as the fraction of hollandite phase in the designer waste form increased while the total fractional Cs release was greater from the samples with greater Cs content. The leach testing results are not intuitive and reaffirm recent suppositions that Cs release from multiphase ceramic waste forms is not well understood. However, designer waste forms provide a method to pursue reproducible phase assemblages that can be used to help elucidate the chemical durability of these complex multiphase materials.
